# The assessment and management of pain in patients with dementia in hospital settings: a multi-case exploratory study from a decision making perspective

**DOI:** 10.1186/s12913-016-1690-1

**Published:** 2016-08-24

**Authors:** Valentina Lichtner, Dawn Dowding, Nick Allcock, John Keady, Elizabeth L. Sampson, Michelle Briggs, Anne Corbett, Kirstin James, Reena Lasrado, Caroline Swarbrick, S. José Closs

**Affiliations:** 1 School of Healthcare, University of Leeds, Leeds, UK; 2 Columbia University School of Nursing, New York, NY 10032 USA; 3 Center for Home Care Policy and Research, Visiting Nursing Service of New York, New York, NY USA; 4 School of Health and Life Sciences, Glasgow Caledonian University, Glasgow, UK; 5 School of Nursing, Midwifery and Social Work, University of Manchester, Manchester, UK; 6 Marie Curie Palliative Care Research Department, Division of Psychiatry, University College London, London, UK; 7 Centre for Pain Research, Leeds Beckett University, Leeds, UK; 8 Wolfson Centre for Age-Related Diseases, King’s College London, London, UK; 9 School of Health, Nursing and Midwifery, University of the West of Scotland, Paisley, UK

**Keywords:** Dementia, Pain assessment, Pain management, Aged, Hospitalization, Decision making, Qualitative research

## Abstract

**Background:**

Pain is often poorly managed in people who have a dementia. Little is known about how this patient population is managed in hospital, with research to date focused mainly on care homes. This study aimed to investigate how pain is recognised, assessed and managed in patients with dementia in a range of acute hospital wards, to inform the development of a decision support tool to improve pain management for this group.

**Methods:**

A qualitative, multi-site exploratory case study. Data were collected in four hospitals in England and Scotland. Methods included non-participant observations, audits of patient records, semi-structured interviews with staff and carers, and analysis of hospital ward documents. Thematic analysis was performed through the lens of decision making theory.

**Results:**

Staff generally relied on patients’ self-report of pain. For patients with dementia, however, communication difficulties experienced because of their condition, the organisational context, and time frames of staff interactions, hindered patients’ ability to provide staff with information about their pain experience. This potentially undermined the trials of medications used to provide pain relief to each patient and assessments of their responses to these treatments. Furthermore, given the multidisciplinary environment, a patient’s communication about their pain involved several members of staff, each having to make sense of the patient’s pain as in an ‘overall picture’. Information about patients’ pain, elicited in different ways, at different times and by different health care staff, was fragmented in paper-based documentation. Re-assembling the pieces to form a ‘patient specific picture of the pain’ required collective staff memory, ‘mental computation’ and time.

**Conclusions:**

There is a need for an efficient method of eliciting and centralizing all pain-related information for patients with dementia, which is distributed in time and between personnel. Such a method should give an overall picture of a patient’s pain which is rapidly accessible to all involved in their care. This would provide a much-needed basis for making decisions to support the effective management of the pain of older people with dementia in hospital.

**Electronic supplementary material:**

The online version of this article (doi:10.1186/s12913-016-1690-1) contains supplementary material, which is available to authorized users.

## Background

Pain is common in older adults, affecting one third of people living in the community [1], and representing a considerable gap in treatment, particularly in acute hospital settings. In general, pain management in older people is a complex challenge. Studies in several countries (e.g. Australia [2, 3]; Canada [4]; Brazil [5]; and the United Kingdom (UK) [6]) have reported sub-optimal management, with limited pain assessment, a lack of documentation in healthcare settings, and longer waits for older people to receive analgesia. The majority of published research into pain management in older adults has been carried out in community or home care settings and relatively little is known of how pain is assessed or managed in acute settings [2, 3, 7].

Dementia affects more than 35 million people worldwide, and this figure is set to rise [8]. The condition is characterised by progressive decline in cognition, function and communication, and is often further complicated by co-morbidities and neuropsychiatric symptoms such as agitation and aggression [9]. These factors combine to introduce a critical challenge in the assessment of pain [10]. Studies have shown that up to 50 % of people living with dementia regularly suffer from some degree of pain [11], and there is a growing amount of evidence that pain is undertreated for people with dementia compared to matched controls [12, 13]. Evidence from studies of community or home care settings indicates that identification and management of pain in this patient group is inconsistent and less-than-optimal, particularly in people who may not be able to articulate the presence or intensity of pain through self-report. Poor pain control may lead to an increase in functional decline, slow rehabilitation, disturbances in sleep routine, poor appetite, impaired movement and an increased risk of falling [14–16].

In the UK, an estimated 25–42 % of hospital beds are occupied by older people (over 65) with dementia [17–20]. One study reported that 16 % of people with dementia admitted to hospital were experiencing pain while at rest and 57 % had pain on movement on at least one occasion. In 16 % of cases this pain persisted throughout the admission [20].

An acute hospital ward may be a disorienting and distressing environment for a person with dementia due to heightened/un-escapable noise, bright lighting and unfamiliar staff and surroundings. Poor pain control in the context of this environment is associated with neuropsychiatric symptoms, particularly aggression and anxiety [20]. These symptoms affect over 75 % of people with dementia admitted to acute hospitals and can increase the risk of mortality and cognitive decline [21]. Neuropsychiatric symptoms are particularly challenging for clinical staff to manage, and are often associated with sub-optimal care or inappropriate prescriptions of antipsychotic medications [22]. Consequently, people with dementia are at higher risk of adverse events during their hospital stay [23] and are more likely to spend an extended time in hospital compared to their cognitively healthy counterparts [17, 24, 25].

There are significant challenges for healthcare professionals and clinicians in evaluating pain experiences in people with dementia, primarily due to their patients’ difficulties in recall, interpretation, identification and response to pain. Impairment to memory and insight often leads to pain being reported only at the point of pain being experienced ‘in the here and now’ [26]. Furthermore, behavioural signs of pain may be altered in unexpected ways in a person with dementia; for example, the person may withdraw themselves physically or emotionally, or may become quiet and still [27]. There is currently no single reliable mechanism or method for understanding how pain presents in someone with dementia, particularly due to the subjective nature of pain as an experience. It is important to stress that recognising absence of pain is of equal importance in assessment, in order to avoid the unnecessary use of pain medications with associated side effects, such as an increased risk of falls [28].

Recognising pain in people living with dementia has been described as a “*guessing game*” by some healthcare professionals [29] [p5]. A number of qualitative studies have highlighted that clinicians often use intuitive approaches to the assessment and management of pain. Nurses often report knowing by a ‘feeling inside’ that a patient is in pain [30, 31]. A number of pain assessment tools have been designed to attempt to systematise this intuitive process, making explicit the information or evidence (‘pain cues’) used. These include both verbal intensity rating scales and observational tools for use with patients with dementia, and there are currently a large number available to support pain assessment in this patient population [32]. Recommendations published by the World Health Organisation (WHO) provide a ‘Pain Ladder’ which uses structured assessment to guide clinical decisions in the selection of treatments for pain [33]. Pain assessment tools and the WHO Pain Ladder are decision support tools to assist in recognising, assessing and managing pain in patients with dementia. However, they are not routinely used in practice, nor implemented within a decision theory framework.

This study hypothesised that a rigorously developed decision support tool could help clinicians, carers and people with dementia by improving pain assessment and management in acute hospital settings. To inform the design of such a tool, an exploratory study was conducted to understand how pain is currently recognised, assessed and managed among patients with dementia in representative acute settings in the UK, through the lens of decision-making theory. This paper reports the findings of this exploratory study and discusses the implications for decision support and improvement in clinical practice.

## Methods

### Aims and objectives

The overall aim of this study was to investigate how health care professionals and others recognised, assessed and managed pain in patients with dementia in a range of acute settings. This was to provide the basis for the development of a decision support tool to improve the management of pain for this population.

The study addressed the following research questions:

1. What information is currently elicited and used by clinicians when detecting and managing pain in patients with dementia in acute hospital settings?
2. What is the existing process of decision making for detecting and managing pain in patients with dementia in acute hospital settings?
3. What is the role (actual and potential) of carers in this process?
4. What is the organisational context in which health care professionals operate, with regard to this decision making process?

### Theoretical framework

Pain is multidimensional, consisting of sensory, cognitive, affective and social components. The focus of this study was physical pain (acute and /or chronic), though we acknowledged that pain and emotional distress are closely linked since distress may exacerbate pain symptoms and vice versa. Pain experiences are associated with a multiplicity of factors which are unique to each individual and even in the absence of cognitive impairment, they are very difficult to communicate meaningfully to other people [34, 35].

We conceptualised pain assessment and management as involving decision making processes, such as the accurate interpretation of the patient’s pain experience (an *assessment or judgement*), and taking appropriate actions to ameliorate the pain (making treatment *decisions*) (something we discuss more in depth elsewhere [36]). There are a variety of theories of judgement and decision making [37]. A common central element is the existing information that supports judgements and decisions. This includes the type of information, how it is gathered and where it is found. For example, in the hypothetico-deductive reasoning model of decision making [38, 39], individuals process information to make a judgment, defined as ‘an assessment between alternatives’ [40]. Information is gathered through ‘cue acquisition’, for example, clinical information about the patient such as patient’s verbal reports of pain or observation of their behaviour. Hypotheses are then generated to explain and interpret the information and more information is gathered if needed until a hypothesis is chosen which is supported by the majority of the evidence or information.

**Fig. 1:**
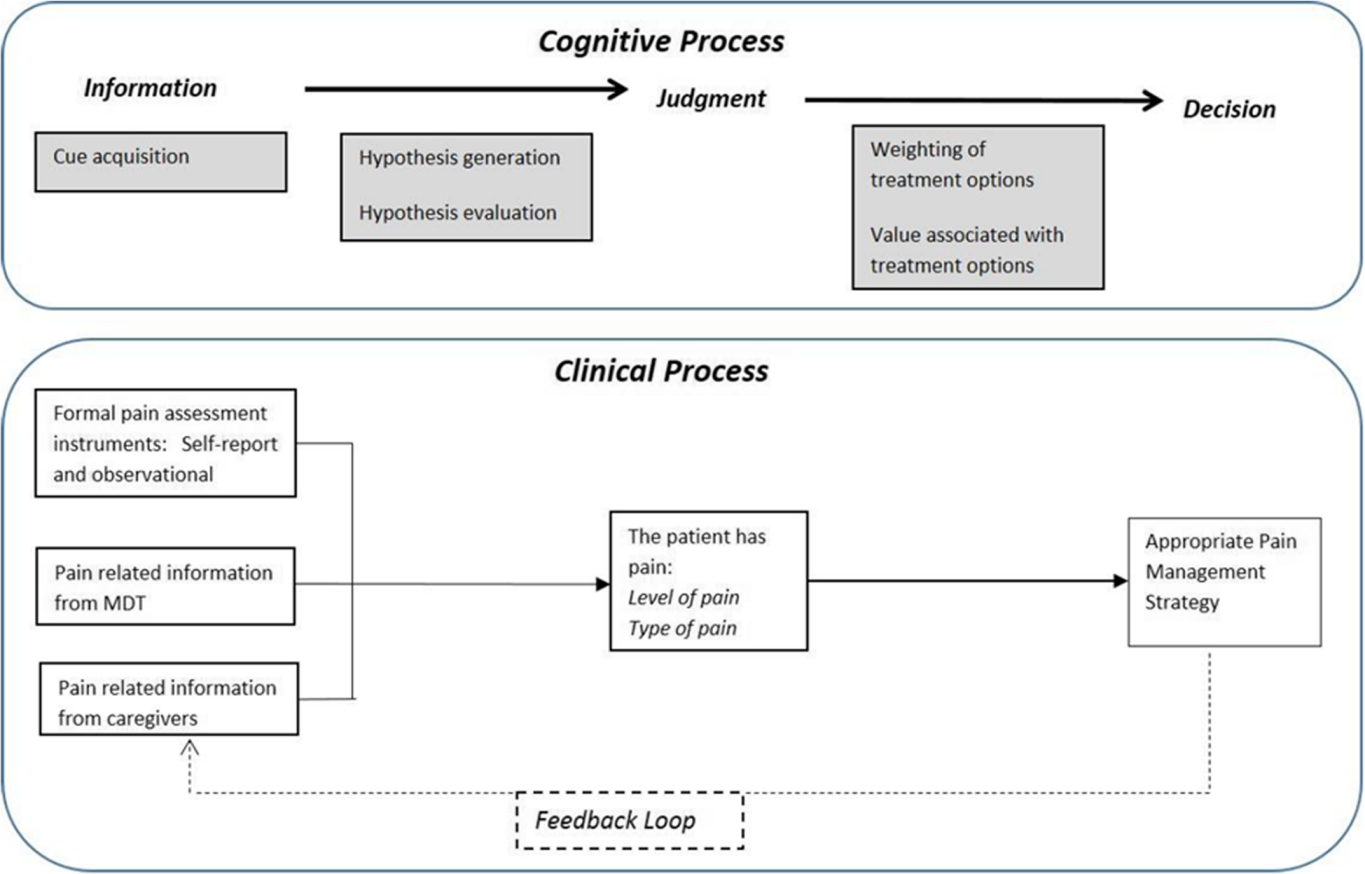
Correspondence between cognitive and clinical process for the recognition, assessment and management of pain. Pain assessment and management involve decision making processes: the interpretation of the patient’s pain experience (an assessment or judgement), and taking appropriate actions to ameliorate the pain (making treatment decisions)

In investigating how health care professionals and others recognise, assess and manage pain in patients with dementia, the research design and research instruments for this study were focused on information (types and availability of information sources, clinicians’ information needs, and methods of recording information), individuals’ processes of perception, judgement, and decision making, as well as any tools used in the process and documentation of a patients’ pain. A model of judgement and decision making focused on linear processes (Fig. 1) was used to guide data collection.

### Design

This study was a multiple case site study with embedded units of analysis (individuals, wards and organisations), approached with ethnographic methods. Case studies involve an empirical design which focuses on describing phenomena within their real life context [41] and appropriate to exploratory objectives.

### Setting

Four case sites (NHS hospital trusts, each with one or more hospitals) were sampled to provide varying settings for acute care: one in the south of England, two in the north of England and one in Scotland. One of the four organisations used electronic patient record systems while the others used paper for medical and nursing notes. Criteria for sampling included type of hospital (tertiary referral centre/secondary care) and type of service provision available to health care professionals in the hospital (e.g. a specialist pain management team, dementia outreach team). In each site two wards were initially selected for data collection, with additional wards approached where access to participants was found particularly challenging. The selection was theoretically driven to ensure that there was representation from a variety of clinical settings in acute care where patients with dementia may be cared for (e.g. orthopaedic, acute medicine, care of the elderly) across the sample as shown in Table 1. This approach was used to ensure a detailed comparative overview would be derived of how pain is currently detected and managed in patients with dementia in a wide range of acute care settings.

**Table 1:** Types of ward included in each Case Site

Case site	Types of ward/medical speciality		
H1	Vascular surgery	Care of the elderly	
H2	Medicine for the elderly	Continuing care	
H3	Stroke rehabilitation	Elderly medicine (3 wards)	Surgery
H4	Surgical/orthopaedic	Acute medical admissions	

### Data collection

Data collection was undertaken by research fellows at each of the four sites. Data were collected at three of the sites (case sites H1, H2, H4) by one researcher each (VL, NG, KJ), and by two researchers in turn in case site H3 (SC, CS). In each case site a variety of data collection methods were used to provide multiple sources of evidence for addressing the research questions. Non-participant observation of health care professionals (HCPs) and health care assistants (HCAs) interacting with patients who have dementia was used to identify how information appears to be identified and elicited in order to detect and manage pain, and the care processes that currently take place. This included observing patients at bedside, and a focus on how and where pain was discussed and documented, interactions between HCPs/HCAs, patients and carers, interactions between members of the multi-disciplinary team (MDT) and availability of resources such as pain specialist services. An observation protocol derived from the theoretical framework was used to guide data collection (see Additional file 1).

Semi-structured interviews lasting approximately 15–60 min were carried out with staff (HCAs, nurses, doctors, other members of the MDT) and patients’ family members (‘carers’). Topic guides were used flexibly, with the freedom to explore any other relevant issues specific to the site (see Additional files 2 and 3). For the most part these focused on exploring people’s perceptions of how pain was detected and managed in each of the wards, how carers were involved in the process, how the process may be improved and what an effective decision support tool would look like (e.g. format, content, resources). Interviews were recorded and transcribed verbatim, with the exception of those conducted in case site H3 which were recorded using handwritten notes.

We looked for any existing policies and procedures in place in the unit and/or organisation that were specifically focused on the detection and management of pain. We also audited patients’ medical and nursing notes for documentation of pain assessment, action taken, pain reassessment and records of prescribed analgesia.

At each site data collection ended when the research team assessed that saturation had been achieved. This was the point at which no new understanding relevant to the research question was being gained. Data were collected between May 2013 and July 2014.

### Participants

Eligible participants were over 65 years of age with a recorded diagnosis of dementia. The degree and type of dementia, and presence of pain were not recruitment criteria, as we were interested in whether and how *potential*, as well as *actual* presence of pain was addressed by staff in the wards. However, patients in the wards we sampled were likely to have undergone medical procedures, or recovering from falls, for example, and it would be highly likely that pain was being experienced.

The sampling of staff and carers for interviews included all members of staff caring for patients in the wards included in the study, in addition to managers and specialists of relevant hospital services. Carer interviews were limited to the family of the patients participating in our study.

The number and length of observation or interviews required to provide an adequate overview of ward-based activities was governed by a notional guide; reaching a specified target (number of participants) was not our concern (actual figures are shown in Table 2). Our research was informed by the principles of theoretical sampling and theoretical saturation [42], rather than those usually found in quantitative sampling. Initially our sampling was done purposefully, on the basis of the aims of the research and the availability of cases (patients who consented). As data collection progressed, we begun the analysis and this informed our further data collection activities.

**Table 2:** Data collection at each case site - Observation of patients

	Case study site				Total
	H1	H2	H3	H4	
Patients Observed (number)	8	7	9	7	31
Mean patient age (range)	83 (77–87)	84 (75–93)	88 (79–99)	85 (75–94)	88 (75–99)
Patient Gender	Male = 1	Male = 2	Male = 4	Male = 4	Male = 11
	Female = 7	Female = 5	Female = 5	Female = 3	Female = 20
Observation time (approximate, number of hours)	71 h	45 h	22 h	32 h	170 h
Time in the field (approximate, number of hours)	161 h	167 h	73 h	85 h	480 h

### Ethics, consent and permissions

Ethical approval was obtained both in England (NRES Committee Yorkshire & The Humber - Leeds West - REC reference: 12/YH/0363) and Scotland (Scotland A Research Ethics Committee, Edinburgh - REC Reference 13/SS/0006). The process to recruit patients was informed by the Mental Capacity Act 2005 and the Adults with Incapacity Act (Scotland) 2000; it included written consent by patients or agreement from a carer consultee, patients’ capacity assessment to consent, consultation with staff and assent of carers [43]. Interviewees gave their written consent and were informed of the audio-recording. The NHS trusts participating in this study granted access to the researchers, who complied with local requirements for data collection. Data were anonymised at the time of data collection.

### Data analysis

Data for analysis consisted of verbatim transcripts of observation sessions, field notes of medical and nursing records, notes and transcripts of interviews. Data were indexed using NVivo (v10) qualitative analysis software, and subjected to thematic analysis. Both inductive and deductive approaches were applied, with dimensions of decision making (information/pain cues and documentation; judgment/pain assessment; decision/pain management) providing initial categories for indexing data, but with a variety of other themes (e.g. about the context of care) emerging from the data.

The strategy for the multi-site qualitative data analysis emerged through a process of team meetings, sharing of documents and reflections among the interdisciplinary team of researchers and investigators. It became apparent that a large number and varied range of themes were emerging from the analysis but it was agreed that a focus on dimensions of decision making was necessary to answer the research questions. The senior research fellow in the team (VL) led the multi-site analysis, which was then discussed with the research team and verified by other researchers (CS, RL, KJ). Transcripts were scrutinised to identify themes or categories, which were used to code the data. Subsets of the dataset were coded by three of the researchers (VL, KJ, RL). The senior research fellow checked a sample of each subset to verify consistency in the analysis. Data in each theme were examined to look for negative cases or contradictory findings [44]. To increase transparency in the analytic process, team meetings of the researchers from each of the sites convened with the project analysis group (including also SJC, NA, JK, MB), on a regular basis during and after the data collection period. This ensured consistency between the four sites. Emerging themes were compared, contrasted, discussed within the group and with the wider project team until a consensus was reached.

## Results

### Participants’ characteristics

Thirty-one patients with dementia participated in the study and were observed for a total of 170 h at bedside. Fifty-two health care staff and four carers were interviewed (Tables 2–3). Among the staff interviewed across the four case sites were seven HCAs, thirty-one nurses (staff nurses, charge nurses, clinical nurse specialists), three doctors in training, five medical consultants, a pharmacist, a physiotherapist and four clinical educators. Ward management was informed that all members of staff were invited to take part in interviews, but there were challenges in finding time when they would be available and researchers tried to minimise disruption to normal work activities.

**Table 3:** Data collection at each case site – Interviews (number of participants)

	Case study site				Total
	H1	H2	H3	H4	
Interviews with staff	24	13	7	8	52
Interviews with carers	1	3	0	0	4

Patients included in the study had a mean age of 88 years (range 75–99). A number of challenges were encountered in recruiting patients for all case sites. Among them was the lack of a documented diagnosis of dementia in ward notes despite strong indications that the condition was present, and many patients had no available carer.

### Themes emerging from qualitative analysis

The analysis identified four over-arching themes which are discussed below; *communicating pain with dementia*, *carer-clinician communication*, *trials with therapy* and *putting a picture together*.

#### Communicating pain with dementia

In assessing and managing a patient’s pain, the guiding principle used by staff was to rely on self-report (the patient telling about their pain), with the patient being the main information source.

However, for many of the patients observed in the study, communication barriers of various sorts hindered a patient’s ability to provide staff with information on their pain experience. These included: issues related to language and cognitive impairment, the impact of patterns of work on a patient’s ability to communicate, and issues of trust and familiarity. We describe and discuss each of these aspects below.

##### Language and cognitive impairment

Patients with severe dementia showed significant communication difficulties. Interviewees explained that questions about pain should be rephrased to account for this. For example, when questions were asked for the purpose of gathering and recording a pain score in a form structured with the three options ‘mild, moderate and severe’, these were to be translated into words patients could understand, taking into account the ability of each individual:

*somebody might have no concept of what moderate means, for example (nurse specialist, H1).*

Patients appeared to be using various gestures, postures, bodily movements, behavioural prompts, metaphorical expressions and a combination of these in what was interpreted as an expression and communication of pain. Data from interviews suggested that nurses and clinicians also looked at physical and behavioural signs to understand patients’ pain. One interviewee commented that the identification of these non-verbal communication cues depended largely on staff skills, experience, knowledge and perceptions and added that *‘we need to get staff to think differently’* (nurse, H3).

Some patients made use of metaphorical expressions to communicate their experience of pain. For example a patient explained the pain in her knee as “*It’s murder, it’s awful. For quite a while it’s alright and then suddenly is murder*” (field notes, H1) and another patient expressed headache as “*red hot*” (field notes, H3). An interviewee suggested that ‘*it is not always what is said, but how it is said*’ (nurse, H3) that gives a cue of a patient’s pain.

##### Patterns of work, time, location and division of labour

The challenges raised by cognitive impairment in patients were compounded by the organisational context and time frames of staff interactions with patients. Organisational routines and staffing numbers meant that the majority of patients’ encounters with staff during their hospital stay were brief, sometime extremely brief and less frequent when with more senior staff members. One patient said, ‘*they are always dashing*’ (field notes, H1), or in the words of a carer, ‘*it’s just a fleeting glance, they talk to them and off they go*’ (carer, H2).

*as an FY1 [Foundation Year 1 doctor], I’m only seeing the patients for a few minutes at a time then the rest of the time looking at their investigations or trying to organise things, I don’t usually, I wouldn’t say that I actually get to know the patient as a person […] (foundation doctor, H2)*

Furthermore, given the organisation of staff work over rotas and shifts, a patient’s communication about their pain was not always with the same member of staff but may have involved several different HCAs, nurses and doctors. There was awareness among staff that patients with dementia would need more time than usual to communicate pain. For example a local Pain Dementia Care Plan guidance (H1) did include instructions for nurses and HCAs to give enough time to the patient to communicate, but in practice this was a matter of minutes. Indeed in interviews the lack of time was often voiced by the nurses as a concern.

*there’s not enough of us, and we just haven’t that time (staff nurse, H1)*

*patients shouldn’t go a long period of time without their pain being reassessed […] certainly you’ve got to go back, it does recommend half an hour, but as I said, it’s quite difficult to get back within that period of time (senior charge nurse, H2)*

These brief encounters required the patient to be ready to answer questions and to recall their pain experience with little or no forewarning. Moreover, these opportunities at times occurred while patients were otherwise engaged in eating or sleeping, or when they were not prepared to discuss pain.

Patients were directed to use a call button at bedside (a ‘buzzer’) to request assistance. Patients with more severe dementia appeared not to recognise the purpose of the buzzer or forgot it was there, thus severely limiting their ability to communicate pain. Also calls for help, including those using a buzzer could not always be answered immediately, leading to distress and confusion for the patients concerned. Some patients seemed to have no memory of having used the buzzer when a staff member arrived. In other cases patients expressed a disinclination to use the buzzer and disturb busy staff, or they did not know what the buzzer was ‘for’, thus rendering it unhelpful to the person with dementia. Cases were also recorded where patients verbally reported pain, but at a time when there were no staff members present. During bedside observations it became apparent that it was necessary to be in close proximity to the patient in order to communicate, and in the same bay or at bedside, as patients rarely left their bed or chair. At times patients’ needs surfaced out of background noise or while the staff were in the room with other patients and patients were able to attract their attention through verbal communication or behaviour. However, this was not always the case and some patients were alone and without interaction for relatively long periods. Many of the ward routines such as note keeping and handovers took place away from the bedside, thus minimising a clinician’s opportunity to communicate with the patient during periods of alertness, or to detect subtle changes in expression and engagement.

##### Trust and familiarity

Clinicians were aware that relationships of trust and familiarity were important for a patient to communicate their pain. One Elderly Medicine Consultant explained: “*if you feel that somebody cares about you then I’m sure it makes it easier to express it if you’ve got pain”* (medical consultant, H1), and a student nurse of HCA’s background stated, “*if you are connected with your patient, if you, they know you and they trust you, if you build that rapport, that itself will allow you better access to how their pain is*” (student nurse, H4). However it seemed to the researchers that establishing these relationships was not facilitated by the brief time frames available for communication and patterns of interaction.

#### Carer-clinician communication

Relatives, visitors and carers represented an important information source in the recognition, assessment and management of pain. These individuals have been described as ‘a hidden workforce’ (as suggested in [45]). One ward sister explained the reasons for involving carers in the process of pain recognition, assessment and management in terms of ‘how well’ the staff know a patient compared to carers, carers’ ability to communicate on behalf of the patient, and how HCPs would be guided by carers in the management of their loved one’s pain.

*[carers] would know the patient much better than we would and they would be able to assess whether [the patients with dementia] they’re in pain or how they’re feeling, and communicate with us. So yes we often ask for input from the carers or the family members to guide us in how we’re managing the patients(deputy sister, H4)*

Carers were observed as acting as messengers on behalf of the patient, and helped recognise and interpret pain cues. An example of this process is described in a quote from a carer interview:

*my mum has a terrible habit, even though we know as a family, when she needs to go to the bathroom she starts shaking her leg and she was doing that in hospital and she was getting in a panic, she actually was crying, she went in with a really bad urine infection which was causing a lot of pain at the time […] (carer, H2)*

However, the observations conducted in this study showed that the majority of staff communications with relatives were concerning medico-legal reasons of consent or about discharge arrangements, and less about *“the needs of how [patients] are and how we can help them here” (staff nurse, H1).* This observation is strengthened by the lack of documented communication with carers found in patient records. In part this was also due to unavailability of family members in a number of cases (something also supported by findings of an internal audit in case site H3 reported by an interviewee, where more than half of respondents said they did not asked a relative ‘*because the family weren’t there’*). Several carers perceived staff to be occupied, and therefore were reluctant to initiate a conversation, while others expressed that they did not perceive themselves as experts in the knowledge of the person they care about. Some were elderly, and had dementia themselves. Family conflicts, domestic violence, poverty and deprivation seemed also to be complicating factors in some cases.

Staff expressed the belief that there is a need for clinicians to improve communication skills with carers. They explained this as having the ability to elicit the right information from the right relative/carer and assess the trustworthiness of these information sources.

*I hear a conversation at the nurses’ station between nurses about another patient. A nurse spoke with the niece, says the patient is okay at home. The other nurse says the niece may not know, may only see her once a week, to talk with (social worker? assistance?), that they may have a completely different picture. (field notes, H1)*

*the skill that the juniors need to have is in digging out, ferreting out the information that is relevant to a person […] in order for us to make an informed decision […] it’s not just, it’s not just asking the question, “How was your relative before they came into hospital?” It’s really understanding the nitty-gritty of the details, […] of course I don’t have the time, unfortunately, to do all of that for every patient, so […] (medical consultant, H1)*

#### Trials with therapy

The most common pain treatment used in the study sites was analgesic medication. Indeed pain management and pharmacological management of pain often appeared to be one and the same. A limited number of non-pharmacological pain management strategies were used, such as patient re-positioning. Clinicians considered potential side effects of medications, including confusion when making treatment decisions.

In certain wards, depending on the ‘type of patients’, pain medications were prescribed routinely to all patients. For example in a ward in case site H4, an orthopaedic surgery ward, patients received “*pain relief already regularly on their charts for their surgical procedures*” and this prescription was administered “*every six hours*” even if patients did not report pain (staff nurses, H4). This standard acute pain medication prescription followed an established protocol. The prescription was provided by the anaesthetists at the time of surgery. More difficult cases, or when pain was clearly not under control, required escalating out of the routine prescription through contact with a specialist pain team and trialling combinations of different analgesic drugs. In other sites pain medication was administered on an ad hoc basis, depending on the patient’s medical condition. In these cases pain management was commonly approached in a trial and error mode, titrating the dose gradually and assessing the patient’s response.

Importantly, the process of (re)assessment for the purpose of establishing the most appropriate medication encountered similar communication challenges as described above as not all patients were effectively communicating changes in their pain. Judging the level of titration, or the appropriate step in the analgesic ladder, relied on clinicians’ knowledge or ‘sense’ of what the expected pain medication for a given medical condition would be:

*obviously a knowledge of the reason why they’re in hospital and if they’d had a particular surgery, of knowledge of what is happening within the body. […] And how much pain relief somebody would need for that (deputy sister, H4)*

Pain relief medication prescribed to be administered as and when needed (PRN) was usually considered part of this process of titration but could not be used effectively with patients with dementia who would not request additional pain relief.

#### Putting a picture together

Overall, understanding a person’s pain in these acute hospital wards involved investigative work and ‘putting a picture together’ of an individual’s pain (*“we’re trying to build a picture”* - staff nurse, H1). This process required time and availability of information from various sources, including carers, the multidisciplinary team assessment, administration of medication and the patient’s response.

The observation of the context of care and document analysis revealed that patient information was shared through face to face encounters, and written documents such as patient records, medical and nursing notes, transfer reports, checklists, care plans and drug charts. The drug chart was frequently referred to by the majority of team members, and a number of staff respondents in the study stated that they used information available on the drug chart to assess, reassess and review both medication and care plans.

Importantly however, paper-based documentation was fragmented, not easily accessible or poorly organized. The various documents retained and used by different health care professionals were kept ‘in silos’.

*so quite a lot of nursing work has to come down in silos so we have nutrition, tissue viability, falls, dementia, and it doesn’t necessarily speak to each other on paper, which I think we’ve quite siloed risk assessments that it’s then difficult to put together holistically. (nurse manager, H2)*

Comparison of audit data and observations raised a question regarding the quality of data recorded, as suggested in the field notes below.

*The nurse completes the patient’s chart for 15:00. He is still trying to sit up, but the nurse does not help. After the nurse moves away, I [researcher] check the record, which is recorded as confused and alert, despite the patient being asleep since I arrived at 14:53. (field notes, H3)*

In one ward the intentional rounding forms were filled in every two hours, in what appeared to become an administrative, rather than investigative exercise. Staff respondents in the study raised concerns over the large amount of paperwork, some of which they considered redundant.

*when we fill in care plans, we’ve got the specialist assessment [forms] and they say the same things as your care plans (sister, H1)*

We also identified ambiguity in documenting the absence of pain. The interviewees reported the tendency to assume that the patient is not in pain if patients’ pain is not recorded in the documents.

*if there’s nothing written, I suppose I would assume the question hasn’t… has the question been asked? I don’t know, I probably from a personal point of view […], I would have asked it but probably not documented that there was no pain. If I don’t see anything written I would assume that the patient hasn’t complained of pain but I suppose what I can’t say is that they’ve been asked if they’ve got any pain.* (doctor, H1)

No use of decision support tools was observed in any of the settings studied for pain assessment or management. In one site (H1) the Abbey Pain Scale [46] was recommended in the local set of documentation but was not available or appeared not to be known to the staff. Instead the site used a Pain Care Plan, which was written anew for each patient in a loosely structured form, with narrative entries at assessment. This was used with all patients, with or without dementia, and with bare information recorded regarding patient experience of pain or what intervention was used.

One site utilized electronic documentation and at the time of the study it was in the early stages of implementing the Abbey Pain Scale in electronic form. However, no data could be collected regarding this and no staff were observed using it. A manager reported how the tool had been trialed, but “*not well used on the ward*”, that the criteria for using it were unclear and that it ended up been used as a ‘tick box exercise’ (Ward Manager, H3). Wards in case site H2 had recently implemented a generic pain assessment (GPA) form, developed locally, to be used alongside the PACSLAC tool (Pain Assessment Checklist for Seniors with Limited Ability to Communicate) [47]. In the period of our study, for the patients we observed in this site, the GPA was often with a patients’ drug chart, but left blank or only with initial entries without follow-up.

*nurses have so many assessments now to do that [...], they’ve kind of lost their credibility a bit, [the GPA form] it’s just seen as a form and a tick box exercise […] it’s another thing to do and yet they have a hugely frantic day (clinical educator,H2)*

## Discussion

This study explored how pain is currently recognised, assessed and managed in patients with dementia from a decision making perspective. Recognition, assessment and management of pain in patients with dementia in hospital wards involved a number of information sources and individuals at different times and in different places.

The main information source was claimed to be the patient, as the sole individual with insight into their pain. However, cognitive impairment, communication difficulties, the organisation and context of the ward, all contributed to hinder access to this source of knowledge. As also shown in other contexts of care [48], the communication difficulties experienced by patients with dementia were interactional in the sense that they “arise, in part, from their cognitive deficits” but were also “occasioned by, or contingent on, the other’s contributions in interaction” [p13]. The hospital routines and environments generated a modality of interaction that challenged the communication abilities patients with dementia may have had. In fact, a similar finding was discussed about hospital inpatients in general [49], that “the ward social system may also have an effect on patients’ communications […] [in the sense that] the very structure of the work situation discourages patients from communicating clearly” [49] [p108].

**Fig. 2:**
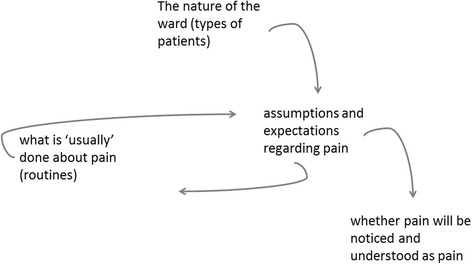
Systemic links between HCAs/HCPs (individuals) perceptions and (organisational) routines. The type of patients in each ward affects staff assumptions and expectations regarding pain and how it should be routinely addressed. In turn this routine affects what can be expected about a patient pain and whether pain may be detected and recognised as pain

The social context of the ward environment also critically shaped health professionals ability to recognise and respond appropriately to a patient’s communication of pain. The time frames for patient interaction, the number of patients on each ward and the expectations set up by the ward routines, all influenced HCPs and HCAs’ ability to perceive, recognise and manage a patient’s pain. Observational studies have revealed the challenges of pain management in acute settings and the barriers to optimal pain relief, such as staffs’ attentiveness to pain cues, interruptions when assessing pain and reconciling varying interpretations of pain from multiple sources [50, 51]. The typology of patients present in a ward – whether the ward was an admission unit, surgery ward, or care of the elderly ward –affected the assumptions and expectations regarding pain and how it should be addressed. This finding echoes that of a study based in a surgical ward unit in the United States where “[p]ain assessment was rooted in a reference typology of clients based on surgical procedure” [51] [p534] and nurses ‘expected’ a ‘certain kind of pain’. The type of patients in each ward influences staff expectations about their pain and how it should be routinely treated. In turn this routine also affects what can be expected about a patient pain and whether pain may be detected and recognised as pain (Fig. 2).

Hypothetico-deductive reasoning [38] posits that clinicians would initially act upon cues given by patients that might suggest pain was present. Being alert and receptive to a pain cue would be the first step in detecting and then managing the patients’ pain. However, the findings from this study showed that cues that were potentially indicative of the presence of pain could be missed, or go unrecognised. Clisset et al. defined ‘missed opportunities’ in dementia care as occasions when “opportunities presented themselves for healthcare professionals to make some connection with the person-with-dementia but they seemed unable or unwilling to do so, often by choosing to end the interaction as quickly as possible” [52]. In this study, missed opportunities manifested in several forms. For example, opportunities to detect and manage pain were missed when the patient was expressing pain but a clinician was not present (co-located) at that moment, when a pain cue was atypical of that which a clinician might usually interpret as pain or when the cue did not seem to merit further investigation as it appeared to fall within acceptable limits.

Analysis of data also revealed organisational routines with an almost exclusive reliance on medication for managing pain. This type of approach is reliant on patients being able to communicate the presence of pain and changes in their pain after administration of medication. However, the communication difficulties in dementia drastically alter the effectiveness of this process. Other pain management interventions, such as repositioning, physiotherapy or physical activity, or patient engagement in meaningful activities, may offer the patients more opportunities to express and communicate their experience of pain and are included in best practice guidelines [53]. Additionally, HCPs aware of the side effects of medications and lack of alternative options for pain reliefs may refrain from asking the patient about their pain, or not pay attention to pain cues, so as not to find themselves then unable to help. As pointed out in [54], when people have a limited action repertoire, the range of issues they notice is more limited (and conversely, the richer the repertoire of action, the wider the issues they notice). Thus it can be argued that reliance on pharmaceutical interventions – and the absence of any other forms of interventions - not only reduces the opportunities for patients with dementia to have their pain more effectively managed, but greatly limits the opportunities for HCPs to assess pain.

### Implications for decision support

**Fig. 3:**
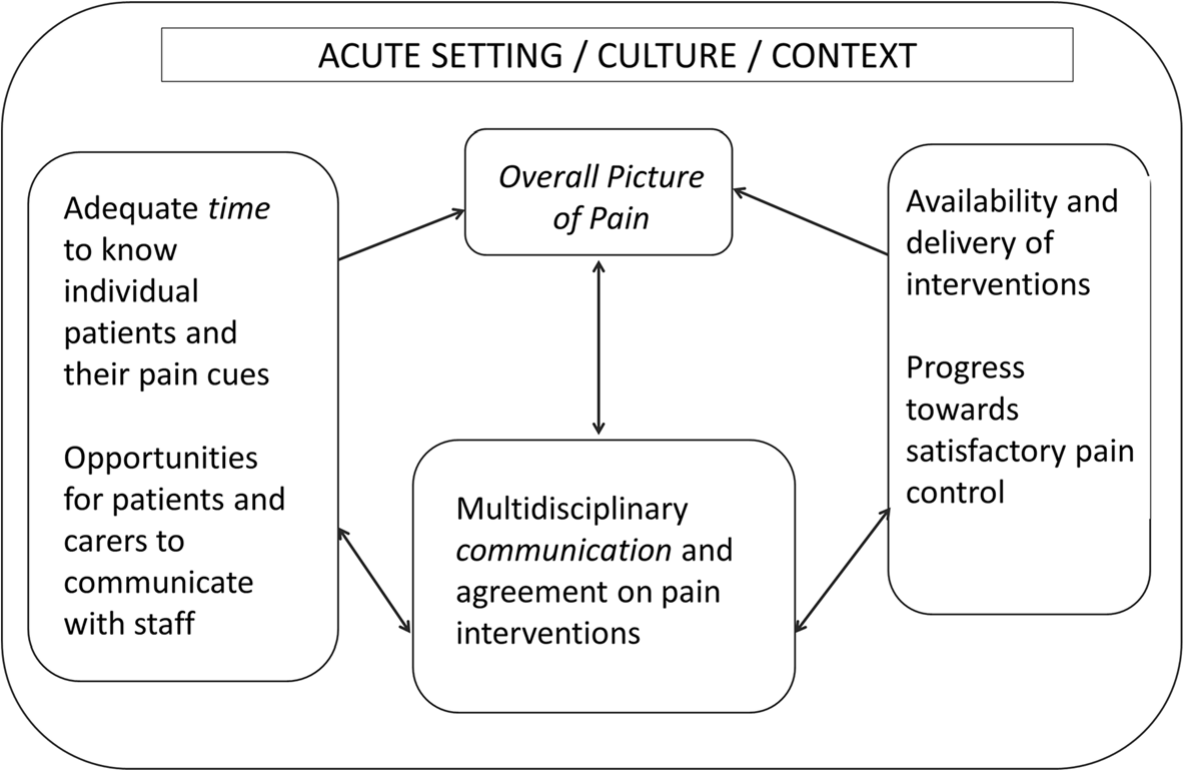
The key elements to obtain a dynamic, patient specific, overall picture of pain. Time, interdisciplinary communication/documentation and the availability of a range of pain management resources are key dimensions for getting to know and recognise pain in patients with dementia. This knowledge is built into a patient-specific ‘picture’ that informs decision making for pain management

The key elements of our findings are shown in Fig. 3, namely the three prerequisites of time, interdisciplinary communication/documentation and the availability of a range of pain management resources. Together, improvements in these areas should facilitate the creation of an ‘overall picture of pain’ to support clinical decision making for optimum pain management. Recognising and assessing pain involves a degree of guesswork, underpinned by medical knowledge and experience (‘what is usual for this medical case’), the most common ‘typologies’ of patients in each ward, and the type of ward (e.g. surgery, or elderly care). When pain is recognised, it is understood through a dynamic sense-making process shared across individuals in the multidisciplinary team in what staff, in this study, referred to as ‘a picture’ of the patient [36]. This key finding has implications for the development of decision support interventions, in that they would need to ensure that they assist with staff identification of pain cues, in part through allowing sufficient time and adequate location to do so. Of note, individuals may have their own unique time-related patterns of pain, related to circadian rhythms, which are known to be disrupted in dementia [55], and the timing of the assessment would need to consider this.

The creation and development of an overall picture of pain, specific for the patient, is both a prerequisite for planning and undertaking a trial of therapy and a result of this trial. In the sites we studied there was a disconnect in documentation between the information about decisions to act (decision to prescribe, decision to administer), the feedback from the action (e.g. outcome of administration of analgesia) and the re-assessment that informs following decisions/actions. This disconnect represented a considerable challenge to the understanding of the patient pain and effectiveness of therapy. It involved on the part of staff, a cognitive process of re-assembling the pieces requiring memory, ‘mental computation’ and time.

Given a ward’s division of labour and organisation of work over shifts, effective documentation is essential for composing this ‘overall picture’ over time, each information item acquiring value when aggregated with and compared to others. Decision tools would need to assist in the integration of the distributed pain information, allowing a central point where the picture of a patient’s pain is created, over time and across individuals. Furthermore, it would have to assist with action and decision taking in a dynamic fashion that supports the trial and error nature of pain management and options for intervention.

The paper based information systems currently in use in the majority of wards in our study for the distributed work processes involved in pain assessment and management do not facilitate the perception of such a holistic ‘picture of pain’. Pain assessment tools are often designed to support and record single instances of assessment, rather than the multiple (re)assessments of a patient’s pain. However, a patient’s pain may change and fluctuate in time and this information on changes and patterns is needed to gain a ‘full picture’ of the patient’s pain. Single pain assessments at one point in time, even if they were done with the aid of one of the many pain assessment tools available, do not provide sufficient information about a patient’s pain; even when repeated they risk remaining as isolated information points because they are not connected in a common, accumulated picture of a patient’s pain, integrating the effectiveness of any pain mitigating care given. Much of the literature concerned with the assessment and management of pain for people with cognitive impairment has focused on the development and validation of observational instruments designed for the purpose of assessment [32]. A number of studies have shown that these tools are often not used in clinical practice, a finding that was supported in our data. We discuss elsewhere [56] the issues with the design of tools that specifically ask for pain intensity scores, and staff difficulties with these. But more generally, a reason for the limited use of observational pain tools in hospital may be that these tools do not facilitate the rapid creation of the ‘overall picture of pain’, and therefore do not assist clinicians with their (distributed) decision-making about pain in this vulnerable group of patients.

### Implications for clinical practice: relationship centred care

A significant part of the information gathering and sense-making process (that would be documented in a ‘pain picture’) is in capturing the pain experiences of the patients themselves. People with dementia who are able to communicate verbally may use a range of metaphors and analogies drawn from their life experience to communicate their pain. Where these vulnerable patients are unable to communicate verbally, effort is required to understand cues unique to each person that may indicate the presence of pain.

When ‘pain pictures’ are informed by the person with dementia’s sense-making of their own situation, these new practices and charts are a potential means of enabling the person with dementia to make their pain understood and documented from a person-centred perspective. To achieve this, it is important to build a relationship with the person with dementia, their carer and other support networks as necessary, in order to elicit self-reports and/or maximise understanding of individuals’ idiosyncratic methods of communication - what is normal for them, and what drugs or other interventions are known to work or not work for them. This process is in line with the gold standard of person-centred care, which is established as a critical element in any dementia care [57, 58]. Such relationships should be forged at the hospital admission point and continue until discharge. Ideally, the HCP-patient relationship should be stable and continuous throughout this time in order to minimise, firstly, the potential for increased environmental disorientation caused by the hospital surroundings and secondly, the missed opportunities that may occur in everyday care.

There is a perennial difficulty of interpreting behaviours which indicate some kind of distress. Information cues (such as patients verbalising their pain, patients’ behaviours, bodily postures, or facial expressions) are often not recognized by staff as indicating the presence or absence of pain, or are misinterpreted as “behavioural problems”. Distress may be due to pain, but it may also have other causes which the patient may be unable to communicate. Familiarity with the patient should enable ward staff to untangle possible causes of distressed behaviours, and to attempt analgesic trials where pain is a likely cause [59].

Finally, the involvement of carers can help clinicians and patients in overcoming communication barriers. At present information provided by carers, when available, is not well integrated in the information system in use or not documented. Increasingly in the UK paper forms are being introduced in hospitals where family members are asked to describe the person’s usual behaviour, likes and dislikes (these are variably known as ‘patient passports’,’10 things about me’, ‘know who I am’, and they are part of a drive to increase awareness of dementia in hospital) but these do not seem to be integrated with a patient medical record. It should be acknowledged that challenges are often encountered in clinicians-carers relationships, and staff perceived need “to manage the family” [45] (e.g. when carers insist that nurses “provide additional or further analgesia when not clinically indicated” [45]). However, a relationship-centred care approach would reframe the carer as part of the identity of the patient, and this would shift the balance from trying to seek out information from carers, to them being actively involved in its creation.

### Policy implications

The challenges associated with pain assessment and management in patients with dementia have underlying contributing factors linked to wider cultural and organisational arrangements at hospital level, organisation of the ward and ward routines, the information systems in use, skill mix and individual clinicians’ beliefs. While this study focused on some of these factors, such as the information systems in use, addressing the root causes of sub-optimal pain care in patients with dementia are likely to require complex interventions in staff education, improvements in resources and organisational infrastructures and change in culture and routine practice. There are valid and powerful arguments to be made regarding the importance of improved infrastructure and education around pain management, particularly within the cost-effectiveness profile of dementia care (with estimated costs in the UK of £20 billion each year [60]). Behavioural symptoms and institutionalisation are key factors in dementia’s large costs to the economy, both of which are closely linked to pain management [61].

### Implications for research

The vast majority of the research concerned with managing pain in people with dementia has taken place in care homes, with most being undertaken in the US, Europe and Australia. Very little research has considered issues encountered in busy acute hospital settings. Furthermore, most research in the field has focussed on the development and validation of observational pain instruments, with less attention paid to the contextual factors influencing their use. Not only should suitable assessment tools be used, but also the relative contributions of factors such as improved use of time, improved multidisciplinary communication and a more varied range of resources for managing pain need to be understood. Realist studies of clinical practice where decision support tools have been implemented could explore the relevance of contextual contributory factors. This should be followed by clinical trials of the effectiveness of the interventions likely to have the most positive impact. There is also a need for research to evaluate integrated approaches to pain management, considering behavioural symptoms, prescription patterns and institutionalisation, in order to embed pain management within the overall treatment and care a person receives throughout their journey with the condition.

### Strengths and limitations

There are few in-depth qualitative studies about pain assessment and management in patients with dementia in hospital settings (e.g. [2, 3]), and to our knowledge no-one has been conducted through the lens of decision making. This theoretical perspective has informed data collection and analysis, which remained focused mainly on dimensions of information and communication of information. Core aspects of the study design were observations at bedside, providing an almost unique opportunity to experience hospital patient care from the patient’s vantage point. The opportunity cost of this approach involved the loss of observation of concurrent activities outside the patient view, several of which could have involved decisions about patients’ pain. However, this was compensated for by additional observations in the ward – for example at nurses’ stations, or doctors’ offices. Challenges in recruitment both of patients and interviewees limited the number of participants in the study, but meant a large number of hours were spent in the ward, for example while waiting for family to visit the patients identified as potential participants. A very small number of carers took part in recorded interviews but conversations took place with all carers who were informed of the study, and the field notes of these brief conversations were included in the analysis. Finally, our findings are mainly based on wards that relied on paper-based information systems (medical, nursing notes, drug charts); one case site had an electronic system in use but we were unable to collect data on how these were used in practice to communicate information about patients. A consultant from a ward in another site that was planning to implement a hospital-wide electronic patient record system expressed the belief that this would facilitate aggregating and sharing information in one place accessible to all, would reduce fragmentation and dispersion of information. The literature instead suggests electronic records may introduce such fragmentation when each piece of information is recorded in a separate screen [62], but we leave this as a question for further research.

Similarly, we have only limited data on clinicians’ views and reasons for not using structured pain assessment tools and further research could be done on this. However, when asked, clinicians may have difficulties making their rationales explicit, and there may be a tendency to blame a general lack of time as the culprit. As suggested in this study, some of the reasons may lie instead in a lack of fit between design of the tool, staff information needs and work practice in the different contexts of care.

## Conclusion

Pain assessment and management are activities that embrace patients’ physiological, emotional, cognitive, and social dimensions. Pain is often described as a private experience but in reality it regularly requires public expression in order to obtain relief. When caring for people with dementia, communication between patients and staff about pain is made more difficult due to the challenges that the progressive cognitive and functional decline present, with non-verbal communication (‘observational pain cues’) becoming more essential and yet more ambiguous. Furthermore, communication occurs within hospital contexts and routines organised mainly around the needs of the organisation rather than those of individual patients. Information about a patient’s pain for the purpose of pain management is generated and constructed through the activity of multiple people at different times; knowledge about patients’ medical condition and expectations about probable pain, inform the times and patterns of interaction, and these affect the way pain is identified and documented, and what is expected and done about pain. The pain assessment and management process is distributed and complex, with mechanisms that are multiple and recursive rather than linear. This complexity may, at least in part, explain why simple tools available to assess pain in patients with dementia are not well used in practice. Future decision support interventions need to take this complexity into account during their development, re-organising frames and quality of time for communication with patients, making a more varied range of pain management interventions routine, and devising tools that bring together information to provide a ‘picture of a patient’s pain’ accessible to all involved, within an overall framework of person-centred care.

## Additional files

Additional file 1: Observation protocol. The research protocol used for observations of patients at bedside. (PDF 156 kb) Additional file 2: Interview Schedule for Staff. The interview guide used in interviews with HCPs and HCAs. (PDF 68 kb) Additional file 3: Interview Schedule for Carers. The interview guide used in interviews with family members of the patients participating in the study. (PDF 67 kb)

## Abbreviations

GPA: Generic pain assessment
HCA: Health care assistant
HCP: Health care professional
MDT: Multi-disciplinary team
NHS: National Health Service
NRES: National Research Ethics Service
PACSLAC: Pain Assessment Checklist for Seniors with Limited Ability to Communicate
PRN: *Pro re nata* (latin), for medicines as and when needed
REC: Research Ethics Committee
WHO: World Health Organisation
