# Supplementary material for: The assessment and management of pain in patients with dementia in hospital settings: a multi-case exploratory study from a decision making perspective
Source: BMC Health Serv Res. 2016 Aug 24;16(1):427. doi: 10.1186/s12913-016-1690-1 (PMC4995653; doi:10.1186/s12913-016-1690-1)
Supplement: Additional file 1: — Observation protocol. The research protocol used for observations of patients at bedside. (PDF 156 kb) [file 12913_2016_1690_MOESM1_ESM.pdf]

The detection and management of pain in patients with dementia in acute care settings: Exploratory Study

Observation Protocol

This observation protocol provides a guide to the types of issues that the researchers will focus on in their field notes. The focus of the observation is the individual with dementia. Observation will focus on identifying if/how pain is assessed and managed by clinical staff, and the behaviours that are being manifested by the individual. Researchers will not be directly observing the provision of direct care that requires privacy (such as washing, dressing, toileting, dressing changes). They will make a note that this has occurred.

Following each episode of care activity/communication the researcher should write a summary paragraph reflecting on the issues that they have noted and comparing this observation to those that have previously been observed.

During the period of observation the researcher will record any interactions that the patient has with clinical staff/carers/other visitors on the ward, making a note of:

- Date, time
- Description of patient: age, sex, presenting complaint
- Duration of interaction
- What is the focus of the interaction (e.g. asking/seeking information from the patient, provision of direct care, administration of medication)
- Details of the interaction (what is said, responses, information given to the patient, behaviour shown by the patient).
- Physical layout of individuals in the interaction (e.g. where are individuals sitting/standing when they interact, how many of them is there).
- Whether or not any pain assessment or management activity has taken place (such as a nurse asking if they are in pain, providing pain medication)

At other times the researcher will be making a note of the patient’s behaviour; what they are doing, whether or not they appear to be in distress, how this is manifesting itself.
