# Supplementary material for: The assessment and management of pain in patients with dementia in hospital settings: a multi-case exploratory study from a decision making perspective
Source: BMC Health Serv Res. 2016 Aug 24;16(1):427. doi: 10.1186/s12913-016-1690-1 (PMC4995653; doi:10.1186/s12913-016-1690-1)
Supplement: Additional file 2: — Interview Schedule for Staff. The interview guide used in interviews with HCPs and HCAs. (PDF 68 kb) [file 12913_2016_1690_MOESM2_ESM.pdf]

The detection and management of pain in patients with dementia in acute care settings: Exploratory Study

Interview Topic Guide: Ward Staff

This interview guide is for interviews with members of the multidisciplinary team, such as doctors, nurses, therapists, and healthcare assistants/support workers.

Before starting, check clinician's understanding of the purpose of the research and that they are happy to participate.

1. To begin with, ask about the interviewee's role and responsibilities, and the types of patients usually under their care
2. Focusing on pain assessment and management in general, ask how is pain recognised, assessed and managed in patients under their care. Invite the interviewee to think of a patient they are caring for at the moment, and to tell how they knew if she was in pain (pain recognition), how they assessed it (pain assessment) and how was it managed (pain management).
   Inquire also on who is responsible for the different aspects of this process.
3. Focusing on patients with dementia, ask whether the process would be any different. Invite the interviewee to think of a patient they are caring for at the moment and relate their answers to this patient.
   Prompt: A patient with dementia may not know how to communicate her pain, or may not remember that the pain is recurrent: how would this affect how their pain is recognised and assessed.
4. Focusing on pain assessment tools, ask about any tools in use in the ward specifically for pain assessment, and if any, for use with patients with dementia.
5. Focusing on the communication and documentation of assessment/management of pain, ask how is activity documented and information communicated.

6. Focusing on the role of carers in the process, ask whether carers are currently involved and how. Invite the interviewee to think of a patient they cared for, and to tell whether the involvement of the carers or relatives changed the recognition, assessment or management of the patient’s pain.
7. Invite the interviewee to reflect on the current assessment/management process and if/how they think it could be improved. Invite the interviewee to reflect on existing tools and think how an effective pain assessment and management tool would look like.

   Prompts: for example in terms of format, content, or information resources.
