# Supplementary material for: The assessment and management of pain in patients with dementia in hospital settings: a multi-case exploratory study from a decision making perspective
Source: BMC Health Serv Res. 2016 Aug 24;16(1):427. doi: 10.1186/s12913-016-1690-1 (PMC4995653; doi:10.1186/s12913-016-1690-1)
Supplement: Additional file 3: — Interview Schedule for Carers. The interview guide used in interviews with family members of the patients participating in the study. (PDF 67 kb) [file 12913_2016_1690_MOESM3_ESM.pdf]

The detection and management of pain in patients with dementia in acute care settings: Exploratory Study

Interview Topic Guide: Carers

Before start check carers understanding of the purpose of the research and that they are happy to participate.

1. Could you tell me how long you have been caring for (name of patient)?
2. Are you also caring for other people (prompt: other members of your family, friends or neighbours)?

   [If yes, explain that all following questions are about (name of patient)]
3. Apart from memory loss, are there any other factors that could impact on hospital staff's ability to manage (name of patient)'s pain/discomfort?
4. How can you tell if (name of patient) has pain or is uncomfortable? What sort of signs do you look for? Have the hospital staff asked you if you can tell if (name of patient) is in pain?
5. How do you try to relieve their pain/discomfort when they are at home? How do you tell if they have worked? Have the hospital staff asked you what you do to help them at home?
6. What actions have hospital staff taken that you think have really helped with their pain/discomfort?
7. How do you think their pain/discomfort has been managed?
8. What do you think could have been done better to help with their pain/discomfort?
9. Do you feel that you have been able to provide information to the hospital staff to help them care for (name of patient) effectively? Why? Could things be improved? How could they be improved?

10. What could be put in place to help you feel confident that (name of patient) has their pain/discomfort managed effectively while they are in hospital?
11. What do you think of the care that they have received while they have been in hospital?
